# Host mitochondrial apoptotic signatures in children with chronic conditions reveal viral modulation of MCL-1 in severe SARS-CoV-2 infection

**DOI:** 10.1007/s11033-026-11885-w

**Published:** 2026-05-09

**Authors:** Emilly Henrique dos Santos, Gabriel Acca Barreira, Mariana Okay Saippa, Lucas Augusto Moysés Franco, Mussya Cisotto Rocha, Kelly Aparecida Kanunfre, Maria Fernanda Badue Pereira, Heloisa Helena Sousa Marques, Thelma Suely Okay

**Affiliations:** 1 Seroepidemiology Laboratory, Institute of Tropical Medicine, School of Medicine, University of Sao Paulo, Sao Paulo, Brazil; 2 Department of Pediatrics, School of Medicine, University of Sao Paulo, Sao Paulo, Brazil; 3 Medical Investigation Laboratory (LIM-36) - Clinical Pediatrics, Hospital das Clinicas, School of Medicine, University of Sao Paulo, Sao Paulo, Brazil; 4 Albert Einstein Israeli School of Health Sciences, Sao Paulo, Brazil; 5 ABC School of Medicine, Santo Andre, Sao Paulo, Brazil; 6 Department of Pathology, School of Medicine, University of Sao Paulo, Sao Paulo, Brazil; 7 Medical Investigation Laboratory (LIM-46) - Microorganism Genomics, Hospital das Clínicas, School of Medicine, University of Sao Paulo, Sao Paulo, Brazil

**Keywords:** Pediatric COVID-19, MCL-1 isoforms, Mitochondrial apoptosis, Biomarkers, Disease severity, Chronic diseases

## Abstract

**Background:**

Although pediatric SARS-CoV-2 infection is usually mild; children with chronic conditions are at increased risk of severe disease. Myeloid Cell Leukemia-1 (MCL-1), a key regulator of mitochondrial apoptosis, is expressed as one anti-apoptotic isoform (MCL-1 L) and two pro-apoptotic isoforms (MCL-1 S and MCL-1ES), but their regulation in pediatric viral infections remains unclear.

**Methods and results:**

We assessed MCL-1 isoform expression by RT-qPCR and circulating protein levels by ELISA in 324 unvaccinated children (179 SARS-CoV-2-positive and 145 SARS-CoV-2-negative), most with chronic conditions. Statistical analyses included parametric and non-parametric tests, correlation analysis, ROC curves, and multinomial logistic regression. All MCL-1 isoforms were upregulated in respiratory viral infections compared with healthy controls. In contrast, SARS-CoV-2 infection showed attenuated MCL-1 transcript induction, suggesting impaired mitochondrial apoptotic signaling, whereas non-SARS-CoV-2 viruses and coinfections induced robust upregulation. Among SARS-CoV-2-positive patients, MCL-1ES predominated, with increased expression in 95% of subgroups and higher levels in boys, adolescents, and coinfected participants. Chronic conditions, particularly cancer, were major drivers of MCL-1 dysregulation. MCL-1 S and MCL-1ES showed good-to-excellent accuracy for predicting mild and moderate disease.

**Conclusions:**

The proposed MCL-1 isoform ratio including MCL-1ES, strongly predicted disease progression, conferring a 6.5- to 7.6-fold increased risk of critical illness, whereas absence of chronic conditions reduced this risk by 94.8%. In addition to chronic medical conditions, this study identified dysregulated MCL-1 isoform balance as a potential determinant of adverse pediatric SARS-CoV-2 outcomes, and supports mitochondrial apoptosis modulation as a host pathway exploited by SARS-CoV-2 and potentially by other viruses with similar immune evasion strategies.

**Supplementary Information:**

The online version contains supplementary material available at 10.1007/s11033-026-11885-w.

## Introduction

MCL-1 (Myeloid Cell Leukemia 1), a member of the BCL-2 (B-Cell Lymphoma 2) protein family, plays a key role in maintaining cellular homeostasis by regulating cell survival and mitochondrial-mediated apoptosis [1]. MCL-1 is essential for the development, function, survival, and longevity of immune cells, and its dynamic expression enables effective immune responses to inflammation and infection [2]. Under physiological conditions, the MCL-1 gene primarily produces the long isoform, MCL-1 L, which has anti-apoptotic properties. This isoform prevents cell death by binding to and sequestering pro-apoptotic proteins such as BAK (BCL-2 Antagonist/Killer) and BAX (BCL-2 Associated X Apoptosis Regulator) [2, 3]. When homeostasis is disrupted, alternative splicing of MCL-1 mRNA favors the generation of two pro-apoptotic isoforms, MCL-1 S (short) and MCL-1ES (extra-short), at the expense of MCL-1 L [4, 5]. Furthermore, post-translational mechanisms can prolong the short half-life of MCL-1 L (approximately 20 min), enhancing its anti-apoptotic function [2, 6].

MCL-1 isoform expression has been well investigated in cancer patients in whom the long isoform of MCL-1 (MCL-1 L) is frequently overexpressed, contributing to tumor cell survival, resistance to chemotherapy, and immune evasion [7, 8]. This upregulation enables malignant cells to evade apoptosis, making MCL-1 a potential therapeutic target. Several hematologic malignancies are particularly dependent on MCL-1 for survival, and a high MCL-1 L/ MCL-1 S ratio indicates suppressed apoptosis. To overcome this resistance, clinical trials are investigating combination therapies that include both BCL-2 and MCL-1 inhibitors. These regimens aim to reduce the MCL-1 L/MCL-1 S ratio, trigger mitochondrial hyperpermeabilization, and promote tumor cell death [9, 10].

While most pediatric patients experience asymptomatic or mild COVID-19, a small proportion develop severe respiratory complications requiring hospitalization, intensive care admission, and may ultimately result in death [11]. The incidence of symptomatic disease in children has shifted over the course of the pandemic, as the emergence of SARS-CoV-2 variants Delta (B.1.617.2) and Omicron (B.1.1.529) was associated with higher pediatric hospitalization rates [12–16].

In Brazil, from 2020 to March 2021, SARS-CoV-2 lineages transitioned from ancestral strains (B.1.1.28/B.1.1.33) to emerging variants, particularly Zeta (P.2) and the more pathogenic Gamma variant (P.1), which became dominant by early 2021 [17, 18]. In children, although most infections remained mild, the emergence of Gamma was associated with higher viral loads, increased transmissibility, and a relative rise in pediatric hospitalizations and severe outcomes, including multisystem inflammatory syndrome in children (MIS-C) [19]. Subsequent SARS-CoV-2 variants Delta and Omicron further increased transmissibility but differed in intrinsic severity, with Delta showing variable impacts across regions and Omicron associated with lower severity but higher case numbers and a reduced incidence of MIS-C [20–22].

During COVID-19, MCL-1 plays a critical role at the intersection of immune dysfunction and viral pathogenesis. Studies investigating interactions between SARS-CoV-2 and human proteins (interactome) have demonstrated significant upregulation of MCL-1 during infection [23, 24]. SARS-CoV-2 appears to exploit host cell survival pathways, including those involving MCL-1, to evade immune responses, and prolong the survival of infected cells [25]. In addition, hyperactivation of immune cells such as macrophages and neutrophils can disrupt MCL-1 regulation, contributing to the cytokine storm characteristic of severe COVID-19 [26–28]. These findings suggest that MCL-1 may represent a promising therapeutic target not only in cancer, but also in the treatment of severe and critical COVID-19 [10–16].

This study aimed to evaluate MCL-1 isoform expression and serum levels of MCL-1 in pediatric patients with chronic medical conditions infected by SARS-CoV-2, and investigate their association with disease severity.

## Materials and methods

### Ethical approval

This research was approved by the Institutional Ethics Committee (CAAE: 30344420.6.0000.0008, April 20, 2020). Informed consent and age-appropriate assent were obtained. Procedures followed the Declaration of Helsinki (October 2024 version).

### Study design

This is a prospective single-center cohort study performed at *Instituto da Criança e do Adolescente*,* Hospital das Clínicas* (*São Paulo*), Brazil, following the STROBE guidelines (https://www.strobe-statement.org/). Clinical trial number: not applicable.

### Participants, inclusion and exclusion criteria, blood sampling

Patients aged 0–18 years who presented with respiratory symptoms such as runny or stuffy nose, sneezing, sore throat, dry or productive cough, difficulty breathing, and/or fever at the Emergency Department of the *Instituto da Criança e do Adolescente*,* Hospital das Clínicas*,* São Paulo*, Brazil, were invited to participate in the study. Enrollment was conducted sequentially after obtaining informed consent. When applicable, informed assent was also obtained. Participants were classified according to their SARS-CoV-2 status determined by RT-PCR (Allplex™ 2019-nCoV assay, Seegene Inc., Seoul, South Korea) on nasopharyngeal secretions. Exclusion criteria were: refusal to participate, confirmed respiratory bacterial infections, patients undergoing chemotherapy, antibiotic treatment or receiving anti-inflammatory drugs to treat acute conditions because these are well-established conditions that can interfere with apoptosis [56]. Nevertheless, residual confounding factors could not be entirely excluded and should be considered when interpreting the results. Blood sampling took place from April 2020 to March 2021. For each participant, two blood samples were obtained at the time of the Emergency Department visit: one in a tube containing EDTA, and another in a tube with a separator gel, without anticoagulant.

### Control groups

As blood sampling from healthy children and adolescents in a tertiary care setting such as ours was very limited considering we were experiencing a viral pandemic, control samples were retrospectively selected. The first control group consisted of 77 healthy individuals with ages varying between 18 and 21 years old, whose whole blood samples had been collected and mixed (volume-volume) with the RNAlater reagent (Thermo Fisher Scientific) to preserve RNA molecules in the course of a previous research. After removal of RNAlater, samples were divided into 500 uL aliquots and stored at -80 °C to avoid further thawing-freezing cycles. Although we did not manage to collect samples from infants, toodlers and school-age children, a number of countries like the United States classify people up to the age of 21 in the pediatric population [57–60]. This first control group was used to ensure that the three RT-qPCR to detect and quantify MCL-1 isoform expression, as well as the RT-qPCR for GAPDH (glyceraldehyde-3-phosphate dehydrogenase), were adequately set up, i.e., their relative expression did not increase in healthy individuals (average fold change should be < 2.1).

The second control group was composed of 23 healthy school-aged children and adolescents (7–14 years) whose serum samples had been obtained in a previous study, aliquoted and stored at -20 °C without prior freeze-thawing cycles. These samples were used for total MCL-1 protein quantification by a commercial ELISA.

All control samples were collected before the COVID-19 pandemic.

### Whole blood samples processing

Immediately after collection at the Emergency Department, a whole blood aliquot (500 µL) was transferred into 2.5 mL of RNAlater reagent (Thermo Fisher Scientific) to ensure complete penetration and rapid inactivation of RNases. The mixture was homogenized thoroughly and incubated overnight at 4 °C to guarantee full equilibration and adequate penetration of the reagent into all cells. After this incubation, samples were stored at -80 °C.

### Non-SARS-CoV-2 respiratory viruses detection

In addition to the SARS-CoV-2 testing by RT-qPCR, nasopharyngeal swabs were screened for other 19 respiratory viruses Luminex xTAG RVP FAST v2 assay (Luminex Molecular Diagnostics, Canada) according to the attendant physician request at the Emergency Department.

### Chronic diseases (CDs)

Participants were categorized according to their primary need of medical care in our institution, in four categories: Genetic Diseases like Inborn Errors of Immunity, Cystic Fibrosis, Rubinstein-Taybi, Down and Di George syndromes, among others; Cancer (leukemia, lymphoma, nephroblastoma, central nervous system tumors, liver carcinoma, among others); Major malformations compromising neurodevelopment and feeding; Organ failure (lungs, kidneys, liver) or organ transplant (liver, kidneys and bone marrow).

### Severity classification

COVID-19 severity (mild, moderate, severe, critical) was firstly evaluated at the emergency room, and again 6–8 days after the beginning of respiratory symptoms, according to the participant’s report (in the second assessment, the severity level was established), following a standardized COVID-19 severity protocol which has been applied even after the emergence of SARS-CoV-2 variants Delta and Omicron [55].

### RNA and cDNA preparation

After removal of RNAlater by a rapid high-speed centrifugation followed by removal of the supernatant, RNA extraction used 140 µL of whole blood and the QIAamp Viral RNA Mini Kit (QIAGEN, Germany) following the manufacturer’s instructions. Then, RNA samples were reverse-transcribed with the iScript cDNA Synthesis Kit (Bio-Rad, USA), according to the recommended protocol.

### RT-qPCR development and validation

The development and validation of the four real-time RT-qPCR assays to amplify the three MCL-1 isoforms (L, S and ES) and the GAPDH housekeeping gene used as control required the evaluation of multiple parameters to ensure accuracy, specificity, and reproducibility. Amplification efficiency ranged between 90% and 110%, with a corresponding standard curve slope between − 3.1 and − 3.6 (ideally around − 3.2) and a coefficient of determination (R²) ≥ 0.98–0.99, indicating strong linearity across serial dilutions. Specificity was confirmed by a single, well-defined peak in the melting (dissociation) curve, consistent melting temperature (Tm) across replicates (triplicates in this study), and a single band of the expected size on agarose gel electrophoresis. No amplification was observed in no-template controls (NTCs), excluding contamination and primer-dimer formation. Technical reproducibility was assessed by low variability among replicates (Ct variation ≤ 0.5-1.0). For relative quantification methods such as 2^−ΔΔCt, comparable amplification efficiencies between target and reference genes are essential, and the reference gene GAPDH must exhibit stable expression across all experimental conditions. Additionally, assays should demonstrate a broad dynamic range, proper baseline and threshold settings within the exponential phase, in addition to absence of reaction inhibitors. Collectively, these criteria ensure the reliability and robustness of qPCR data for downstream quantitative and biological interpretation. Detailed assay validation, including amplification efficiency and melting curve analysis, is provided in Supplementary Figures S1-S3.

### RT-qPCR for MCL-1 isoforms

MCL-1 isoforms (L, S, ES) and GAPDH expressions were quantified using previously described protocols (Power SYBR™ Master Mix, Thermo Fisher Scientific, USA), and the following primers: MCL-1L forward 5’- CGGTACCTTCGGGAGCAG-3’ and reverse 5’- CGTCTTCGTTTTTGATGTCCAGT-3’ generating a 175 base-pair (bp) amplification product; MCL-1 S forward 5’- C CTTCCAAGGATGGGTTTGTG-3’ and reverse 5’- ACCAGCTCCTACTCCAGCAACA-3’ generating a 105 bp product; MCL-1ES forward 5’- GAGGGCGACTTTTGGCC-3’ and reverse 5’- CGTCTTCGTTTTTGATGTCCAGT-3 generating a 171 bp product. Cycling conditions for the three MCL-1 isoform amplification and the GAPDH RT-qPCR were: 95 °C for 10 min, then 40 cycles of 95 °C for 15 s and 60 °C for 1 min.

### Relative expression analysis of MCL-1 isoforms

The relative expression of MCL-1 isoforms (MCL-1 L, MCL-1 S, and MCL-1ES) was quantified using the 2^-ΔΔCt method [61, 62]. Briefly, the cycle threshold (Ct) values obtained for each MCL-1 isoform were normalized against the endogenous reference gene GAPDH, generating ΔCt values (Ct_MCL-1 isoform − Ct_GAPDH). This normalization corrects for sample-to-sample variations in RNA input, cDNA synthesis efficiency, and overall reaction performance. Subsequently, ΔCt values from each sample were compared to those of the calibrator group (healthy controls), resulting in ΔΔCt values (ΔCt_sample − ΔCt_control). The relative expression levels, given in fold change, were then calculated as 2^-ΔΔCt, representing the magnitude of upregulation or downregulation of each MCL-1 isoform relative to the control group. For each study group, mean fold change values (average fold) were calculated and used as variables in downstream statistical analyses, enabling comparisons between COVID-19-positive, COVID-19-negative, and control groups. For biological interpretation, fold change thresholds were defined a priori: values greater than 2.1 fold were considered indicative of biologically meaningful upregulation, whereas values below − 0.5 fold were interpreted as relevant downregulation (consistent with a substantial reduction in expression when considered on a relative or log-transformed scale). These thresholds are consistent with commonly adopted practices in RT-qPCR studies, in which approximately twofold changes are regarded as exceeding typical technical variability and therefore more likely to reflect true biological modulation, while − 0.5 fold could indicate a possible true reduction of the target gene expression [61–63].

In the specific context of apoptosis, even moderate shifts in the expression of BCL-2 family members, including MCL-1 isoforms, may have significant functional consequences due to their role in regulating mitochondrial outer membrane permeabilization (MOMP) and cell fate decisions [64, 65]. Thus, the adopted thresholds were considered appropriate to identify biologically relevant alterations in the balance between anti-apoptotic (MCL-1 L) and pro-apoptotic (MCL-1 S and MCL-1ES) isoforms.

### Serum MCL-1 quantification

The Human MCL-1/BCL2L3 ELISA Kit (Thermo Fisher Scientific/ Invitrogen) used in this study is a sandwich assay designed to quantify total MCL-1 protein in serum, plasma, or supernatants, without isoform-specific resolution. According to the manufacturer, the assay detects MCL-1 at the protein level (MCL1/ BCL2L3), and the antigen list includes MCL-1 L, MCL-1 S, and MCL-1ES as members of the same protein family rather than as distinct isoforms. MCL-1 isoforms arise from alternative splicing and share substantial sequence homology; therefore, immunoassays such as ELISA typically use antibodies targeting conserved domains, enabling sensitive but non-isoform-specific detection [33, 34]. Consistently, most commercially available anti-MCL-1 antibodies recognize regions common to all splice variants. As a result, protein-level discrimination among MCL-1 L (~ 38–40 kDa), MCL-1 S (~ 30–32 kDa), and MCL-1ES (~ 24–28 kDa) relies primarily on molecular weight differences rather than antibody specificity [33, 34]. This limitation is further compounded by the scarcity of isoform-specific antibodies for MCL-1 L and MCL-1 S, the lack of antibodies against MCL-1ES, and the high sequence overlap among isoforms, which may hinder the distinction between true MCL-1ES and proteolytic fragments of MCL-1 L [4, 66].

### Statistical analysis

Fold change > 2.1 was arbitrarily considered increased. Significant reductions of fold change were not observed in this study (fold change < -0.5). The expression of MCL-1 isoforms in different groups was given in mean value ± standard deviation. Data distribution was assessed using the Shapiro–Wilk test and was found to be non-normal, therefore, non-parametric statistical tests were applied. Group comparisons were performed using the Kruskal–Wallis test. Spearman’s correlation coefficient for associations, ROC curves to assess predictive value of MCL-1 isoforms, and a multinomial regression analysis to evaluate independent predictors of disease severity. Analyses used GraphPad Prism v8.0 (GraphPad Software Inc., USA), and SPSS software v25.0 (IBM Corp., USA).

## Results

A total of 324 unvaccinated participants with no prior exposure to SARS-CoV-2 were enrolled, including 179 who tested positive for COVID-19 by RT-PCR and 145 who tested negative. All participants tested negative for bacterial pathogens in blood, urine, cerebrospinal fluid (CSF), and other cultures, when assessed. At the time of enrollment, participants were not under chemotherapy, antibiotics, or anti-inflammatory drugs to treat acute medical conditions; however, many of them, particularly transplant recipients, were on immunosuppressive therapy to prevent organ rejection.

### Characteristics of participants

Participants (*N* = 324) were categorized according to COVID-19 status, sex, age, self-declared ethnicity, the presence and type of chronic disease (CDs), presence of non-SARS-CoV-2 respiratory viruses (RVs), and disease severity [27]. No significant differences were found between COVID-19-positive (*n* = 179) and COVID-19-negative (*n* = 145) groups regarding the previously mentioned groups (Table 1).

In the non-COVID-19 group, 80% of participants were non-Hispanic White, 79.3% had chronic diseases, and 29% tested positive for non-SARS-CoV-2 respiratory viruses (RVs), primarily rhinovirus and adenovirus. RV positivity was more common among girls, infants, and those with organ failure or transplants. In the COVID-19 group, 76.5% were non-Hispanic White, 84.9% had chronic diseases, and 38.0% were RV-positive, primarily for rhinovirus and respiratory syncytial virus (RSV). The highest RV detection rates occurred in participants with organ failure or transplants, with a relatively even distribution across sex and age (Table 1).

### COVID-19 severity (Table 1)

Among 179 positives cases, 101 (56.4%) had mild, 20 (11.2%) moderate, 36 (20.1%) severe, 22 (12.3%) had critical disease (12 with MIS-C, 3 infants < 2 years died, all with liver failure and genetic disorders).

### MCL-1 isoform expression and serum levels in COVID-19-positive, COVID-19-negative, and in the negative control group (Table 2)

As expected, the negative control (NC) group (*n* = 77) showed no elevation in MCL-1 isoform expression. In the COVID-19-positive and COVID-19-negative groups, whole blood samples were available for RT-qPCR analysis from 175 of 179 and 138 of 145 participants, respectively. For ELISA, 173 and 142 serum samples were available from the COVID-19-positive and COVID-19-negative groups, respectively, in addition to 23 serum samples from the control group. In both COVID-19 groups, MCL-1 L expression was only mildly increased, MCL-1 S showed a moderate increase, and MCL-1ES exhibited the highest expression levels. Although no overall statistically significant differences were observed between the two COVID-19 groups when analyzing MCL-1 isoform expression and circulating MCL-1 levels, multiple comparison analyses identified significant differences for specific isoforms (*p* < 0.0001) as well as for serum MCL-1 levels (*p* < 0.0001).

Multiple comparisons of fold changes values (MCL-1 isoform expressions) and serum MCL-1 levels (ELISA) were analyzed by the Kruskal-Wallis test. Data are presented as mean ± standard deviation (SD). Statistically significant *p*-values are highlighted in bold.

### MCL-1 isoform expression in subgroups according to disease severity

These parameters were assessed in 175 of 179 COVID-19-positive participants. MCL-1 isoform expression was analyzed according to demographics, presence of other respiratory viruses and chronic diseases (CDs). Severity was stratified into four levels [27] (Table 3). Among other interesting findings, Table 3 shows that severe and critical disease were 6.1 times more common in association with CDs, and 1.8 times less common in RV-coinfected participants.

- **MCL-1 L**: Increased expression (> 2.1 fold change) was found in 45% of 40 subgroup comparisons displayed in Table 3. Increments were highest in moderate disease in girls (3.9 fold), age 3–10 years (7.7 fold), and RV coinfections (5.5 fold). No increase was seen in 0–2 years group.
- **MCL-1 S**: Increased expression was seen in 62.5% of subgroup comparisons. Increases were generally low in severe cases. Exceptions: adolescents (11–18 years) with critical disease; no increases were seen in severe/ critical disease in girls, ages 3–10 years, or in RV-negative or CD-negative participants.
- **MCL-1ES**: Increased expression in 95% of subgroup comparisons. Lowest levels were observed in critical cases except RV-coinfected. Most subgroups showed higher expression in mild to severe vs. critical cases. RV-coinfected subgroup had the highest expression in mild (24.6 fold) and moderate (55.1 fold) cases, dropping in severe (4.4 fold) and critical (8.0 fold) disease, although still significant (> 2.1 fold change).

### Serum MCL-1 levels (ELISA) in subgroups according to disease severity (Table 4)

MCL-1 levels were measured in 173 of 179 COVID-19-positive participants with available serum samples. Results ranged from 8.9 to 25.0 ng/mL across all subgroups. No significant differences were observed between subgroups or across disease severity levels.

### MCL-1 isoform expression and serum levels according to the type of chronic disease (Table 5)

MCL-1 L expression was mildly increased only in cancer patients (3.15 fold). MCL-1 S and MCL-1ES were significantly higher in all CD types (*p* = 0.021), especially in cancer. Serum levels were increased in all CD types, with no differences among them.

### Correlation between serum levels of MCL-1 and MCL-1 isoform expression (Table 6)

While only a weak inverse correlation was found between serum MCL-1 and MCL-1 S in severe cases of COVID-19 (ρ = -0.471, *p* = 0.005), two strong positive correlations were observed when MCL-1 isoforms were compared: ρ = 0.735 with *p* = 0.01 for the comparison MCL-1 S vs. MCL-1 L in severe disease; and ρ = 0.824 with *p* = 0.001 for the ratio comparison, i.e., MCL-1 L vs. MCL-1ES + MCL-1 S, reaching a *p* = 0.001.

### Predictive value of MCL-1 isoforms using receiver operating characteristic (ROC) curves (Fig. 1)

- MCL-1 L: acceptable for mild/moderate disease (AUC 0.70–0.80);
- MCL-1 S: excellent for mild/moderate disease (AUC > 0.90), good for severe/critical disease (AUC 0.80–0.90);
- MCL-1ES: good for mild and critical disease (AUC 0.80–0.90); excellent for moderate disease (AUC > 0.90); acceptable for severe disease (AUC 0.70–0.80).
- The proposed MCL-1 ratio including MCL-1ES expression (MCL-1 L/ [MCL-1 S + MCL-1ES]): good for moderate disease; acceptable for mild or critical cases; poor for severe cases.

### Multinomial regression analysis considering critical COVID-19 as the reference category (Table 7)

Expression of MCL-1ES could predict progression to critical disease (↑ 11-12.7% risk/unit). The proposed MCL-1 ratio including MCL-1ES, strongly predicted progression from mild (556%) or severe (652%) to critical disease (*p* < 0.05). MCL-1 L and MCL-1 S alone were not predictive. Absence of CDs reduced the risk of moderate cases becoming critical by 94.8% (*p* = 0.035).

## Discussion

In this study, we analyzed a cohort of unvaccinated pediatric patients with no prior exposure to SARS-CoV-2, evaluating MCL-1 isoform expression and circulating MCL-1 levels in SARS-CoV-2-positive, SARS-CoV-2-negative, and control participants. By stratifying analyses according to demographics, chronic medical conditions, respiratory viral coinfections, and disease severity, we provide a comprehensive assessment of mitochondrial apoptotic signaling in pediatric acute illness. The overall comparability between groups in terms of age, sex, and clinical characteristics supports the robustness of our findings, although residual confounding from unavoidable clinical factors, such as immunosuppressive therapy, cannot be fully excluded.

Consistent with prior pediatric studies, most SARS-CoV-2-infected children developed mild disease, whereas severe outcomes were concentrated among those with underlying chronic conditions [29, 30]. The lower frequency of severe disease observed among children with respiratory viral coinfections further supports the concept that host-related factors, rather than viral detection alone, are major determinants of disease progression [31, 32].

At the molecular level, our findings demonstrate that acute illness is associated with marked dysregulation of MCL-1 isoform expression. Both SARS-CoV-2-positive and SARS-CoV-2-negative patients exhibited significant upregulation of the pro-apoptotic isoforms MCL-1 S and particularly MCL-1ES, whereas healthy controls showed no such induction [4, 33]. The absence of major differences between viral groups suggests that mitochondrial apoptotic activation represents a common host response to inflammatory stress, largely independent of viral etiology [34]. However, subgroup-specific patterns indicate that SARS-CoV-2 does not merely induce apoptosis but actively modulates apoptotic pathways.

MCL-1 isoforms, generated through alternative splicing, function as a central apoptotic rheostat. While MCL-1 L is anti-apoptotic, MCL-1 S and MCL-1ES promote apoptosis, and their relative balance reflects cellular stress adaptation [4, 34]. Emerging evidence indicates that SARS-CoV-2 regulates this balance through multiple complementary molecular mechanisms. First, direct viral-host interactions have been described, whereby the SARS-CoV-2 nucleocapsid (N) protein stabilizes MCL-1 L via Ubiquitin-Specific Protease 15-mediated deubiquitination, a post-translational modification process, prolonging its half-life and suppressing apoptosis [24]. Second, SARS-CoV-2 infection induces mitochondrial dysfunction and reprogramming of intrinsic apoptotic pathways, which are tightly controlled by BCL-2 family proteins, including MCL-1 [35]. Third, activation of inflammatory and stress-related signaling pathways during infection, such as cytokine-driven responses, oxidative stress, and interferon-mediated signaling, may indirectly influence MCL-1 regulation by modulating transcriptional programs and RNA splicing machinery [36, 37]. These processes are known to alter spliceosome activity and favor the generation of pro-apoptotic isoforms under conditions of cellular stress. Collectively, these findings support a model in which SARS-CoV-2 both directly and indirectly modulates MCL-1-dependent apoptotic signaling through post-translational stabilization of anti-apoptotic isoforms, mitochondrial pathway reprogramming, and stress-induced alternative splicing. The net effect likely reflects a dynamic interplay between host-driven pro-apoptotic responses aimed at limiting infection and virus-mediated anti-apoptotic mechanisms that promote cell survival and viral persistence.

Importantly, we found that MCL-1 isoform dysregulation, particularly increased MCL-1ES expression and the proposed MCL-1 ratio including MCL-1ES, were strongly associated with disease severity and independently predicted progression to critical illness. These findings identify mitochondrial apoptosis as a key component of disease pathogenesis and position MCL-1ES as a promising prognostic biomarker. Notably, these associations appear to extend beyond conventional inflammatory pathways, which have been widely implicated in COVID-19 severity [38].

An important question is how MCL-1 isoform dynamics relate to established biomarkers of inflammation and infection routinely used in clinical practice. Parameters such as the neutrophil-to-lymphocyte ratio (NLR), C-reactive protein (CRP), interleukin-6 (IL-6), and coagulation markers including prothrombin time-international normalized ratio (PT-INR) are well-recognized indicators of disease severity in COVID-19, reflecting systemic inflammation, immune dysregulation, and coagulopathy [39, 40]. In this context, the observed upregulation of pro-apoptotic MCL-1 isoforms, particularly MCL-1ES, is biologically consistent with heightened inflammatory signaling, as cytokine-driven stress responses and mitochondrial dysfunction are known to influence alternative splicing and apoptotic pathway activation [37].

However, unlike these conventional markers, which primarily capture downstream systemic responses, MCL-1 isoform expression may reflect an upstream, cell-intrinsic regulatory layer linking inflammation to mitochondrial apoptosis. This distinction suggests that MCL-1-based markers could provide complementary mechanistic information and potentially improve risk stratification when integrated with established clinical biomarkers. Nevertheless, direct correlations with routine laboratory parameters were not systematically assessed in this study and should be addressed in future investigations.

A central contribution of this study is the identification of a dynamic pattern of MCL-1ES expression across disease stages. We propose a two-phase model of mitochondrial apoptotic response. In early or mild disease, inflammatory stress promotes alternative splicing toward pro-apoptotic isoforms, representing an adaptive host response aimed at limiting viral replication [41]. As disease progresses, this response becomes dysregulated, with a decline in MCL-1ES expression in severe and critical illness. This transition may reflect viral-mediated suppression of apoptosis, now recognized as a key mechanism of SARS-CoV-2 immune evasion, together with exhaustion of apoptotic pathways and sustained inflammatory signaling [42, 43]. In this context, recent studies have highlighted the contribution of integrated cell death programs, such as PANoptosis, in severe COVID-19, further supporting the concept of dysregulated and interconnected cell death pathways [44, 45].

Collectively, these findings suggest that disease severity is characterized not simply by increased apoptosis, but by a failure of coordinated and regulated cell death responses. The observed decline in MCL-1ES expression in severe disease is likely multifactorial, reflecting a combination of apoptotic pathway exhaustion, viral-mediated suppression of pro-apoptotic signaling, and compensatory anti-apoptotic responses, including stabilization of MCL-1 L. These mechanisms are not mutually exclusive and likely coexist during disease progression.

Interestingly, MCL-1 L transcriptional levels remained relatively stable across groups, despite strong evidence supporting its stabilization at the protein level during SARS-CoV-2 infection [24]. This apparent discrepancy likely reflects post-transcriptional and post-translational regulatory mechanisms, underscoring the importance of integrating transcriptomic and proteomic data when interpreting apoptotic signaling [46].

In contrast to isoform-specific transcriptional changes, circulating MCL-1 protein levels were uniformly elevated in hospitalized children, regardless of SARS-CoV-2 status, and did not correlate with disease severity. This dissociation between mRNA expression and serum protein levels is consistent with the notion that circulating MCL-1 reflects generalized immune activation rather than isoform-specific apoptotic signaling [34, 46]. Thus, while serum MCL-1 may serve as a marker of systemic inflammation, it lacks specificity as a prognostic biomarker.

Stratified analyses revealed substantial heterogeneity in MCL-1 isoform expression across clinical subgroups. Respiratory viral coinfections were associated with higher MCL-1ES expression in mild and moderate disease, followed by a marked reduction in severe cases. This pattern parallels the lower prevalence of severe COVID-19 in coinfected children and may reflect viral interference mechanisms, including early interferon responses and enhanced innate immune priming [31, 32, 47]. These pathways may also influence RNA splicing machinery and, consequently, MCL-1 isoform dynamics [37].

Chronic medical conditions also influenced apoptotic signaling. Patients with cancer exhibited the highest levels of pro-apoptotic isoforms, potentially reflecting baseline cellular stress and treatment-related effects, as well as the known dependence of tumor cells on MCL-1-mediated survival pathways [48]. In contrast, children with organ failure or organ transplant showed reduced pro-apoptotic isoform expression, likely due to immunosuppressive therapy and altered immune responsiveness [49]. Age- and sex-related differences were also evident, with boys showing more pronounced alterations in isoform balance and infants exhibiting reduced pro-apoptotic responses, consistent with known differences in immune and mitochondrial function [50].

Correlation and predictive analyses further reinforce the biological and clinical relevance of these findings. Strong positive correlations among MCL-1 isoforms in severe disease suggest coordinated transcriptional regulation under stress conditions. Importantly, ROC and regression analyses demonstrated that MCL-1ES expression and the proposed MCL-1 ratio provide robust discrimination of disease severity and independently predict progression to critical illness.

Beyond SARS-CoV-2, emerging evidence indicates that MCL-1 is a key target exploited by multiple viruses to regulate host cell survival and optimize replication, highlighting its role as a conserved node in host-pathogen interactions [51]. This broader relevance suggests that dysregulated MCL-1 signaling represents a shared pathogenic mechanism across viral infections rather than a SARS-CoV-2-specific phenomenon.

From a therapeutic perspective, modulation of MCL-1 isoform balance represents a promising host-directed strategy. In addition to established MCL-1 inhibitors developed in oncology [48, 52], recent advances have led to the development of novel structure-based inhibitors with improved selectivity and pharmacological profiles [53]. Given that SARS-CoV-2 directly exploits MCL-1 to suppress apoptosis, these approaches may be repurposed to counteract viral immune evasion [24]. However, the therapeutic window is likely narrow, as excessive apoptosis may exacerbate tissue injury, whereas insufficient apoptosis may promote viral persistence and immune escape [42, 54]. Future strategies will require precise and context-dependent modulation, potentially combining splicing regulators, BH3 mimetics, and immunomodulatory therapies to restore apoptotic balance while preserving tissue integrity [46].

### Study limitations

This study has several limitations. First, MCL-1 protein quantification was performed using an ELISA assay that measures total MCL-1 levels without distinguishing between isoforms. Given the high sequence homology among MCL-1 L, MCL-1 S, and MCL-1ES, these measurements reflect aggregate protein levels, limiting interpretation of isoform-specific functional effects. Therefore, integration with transcript-level data is essential to contextualize these findings.

Second, a substantial proportion of patients, particularly transplant recipientes, were receiving immunosuppressive therapies known to influence mitochondrial apoptotic pathways and BCL-2 family proteins. Although this reflects real-world clinical conditions, these treatments may have modulated MCL-1 expression and apoptotic responses. However, modification or discontinuation of such therapies was neither ethically acceptable nor clinically feasible.

Third, while our findings suggest a mechanistic link between SARS-CoV-2 infection and modulation of apoptotic pathways, the observational nature of this study precludes definitive causal inference. Functional assays evaluating apoptosis, mitochondrial integrity, and isoform-specific protein expression in immune cell subsets would be necessary to confirm the proposed mechanisms.

Finally, emerging evidence indicates that SARS-CoV-2 broadly alters mitochondrial and cell death pathways, including apoptosis and PANoptosis [44, 45]. Disentangling virus-specific effects from host inflammatory responses remains challenging and warrants further investigation in controlled experimental models.

## Conclusion

Pediatric COVID-19 is characterized by dysregulated MCL-1 isoform expression, with dynamic shifts in apoptotic balance contributing to mitochondrial dysfunction and disease progression. In particular, MCL-1ES expression and the proposed MCL-1 ratio including MCL-1ES emerged as robust predictors of progression to critical illness, reflecting key host-virus interactions at the level of mitochondrial apoptosis.

Our findings support a model in which SARS-CoV-2 infection does not simply induce apoptosis but actively modulates apoptotic pathways through coordinated transcriptional, post-transcriptional, and post-translational mechanisms. The identification of a two-phase apoptotic response characterized by early adaptive pro-apoptotic signaling followed by dysregulation in severe disease, provides new insight into the pathophysiology of COVID-19 in children.

Importantly, these mechanisms appear to extend beyond SARS-CoV-2, suggesting that MCL-1 represents a conserved target in viral infections and a potential biomarker of host response. From a translational perspective, modulation of MCL-1 isoform balance may represent a promising host-directed therapeutic strategy, although careful consideration of the narrow therapeutic window will be required.

Multicenter and longitudinal studies incorporating functional assays are warranted to validate these findings, refine their clinical applicability, and further explore therapeutic interventions targeting mitochondrial apoptotic pathways in pediatric infectious diseases.

**Table 1:** Distribution of participants according to their COVID-19 status, followed by subgrouping according to age, sex, self-reported ethnicity, respiratory virus coinfection, the presence and type of chronic disease, and disease severity when applicable

Variables	Participants *N* = 324		
	COVID-19 Positive *N* = 179	COVID-19 Negative *N* = 145	*p*-value
*Sex*			
Female	80 (44.7%)	73 (50.3%)	0.331
Male	99 (55.3%)	72 (49.7%)	
*Age group (years)*			
0–2	51 (28.5%)	39 (26.9%)	0.903
3–10	71 (39.7%)	61 (42.1%)	
11–18	57 (31.8%)	45 (31.0%)	
*Self-declared ethnicity*			
Non-Hispanic White	137 (76.5%)	116 (80.0%)	0.461
Mixed (non-Hispanic White and Black)	37 (20.7%)	23 (15.9%)	
Non-Hispanic Black	5 (2.8%)	6 (4.1%)	
*Respiratory viruses*			
Detected	68 (38.0%)	42 (29.0%)	0.160
Undetected	70 (39.1%)	59 (40.7%)	
Not performed	41 (22.9%)	44 (30.3%)	
*Chronic diseases – N (%)*			
Yes	152 (84.9%)	115 (79.3%)	0.188
No	27 (15.1%)	30 (20.7%)	
Type of chronic disease	*N* = 152	*N* = 115	
Genetic diseases	48 (31.6%)	41 (35.7%)	0.700
Cancer	37 (24.3%)	22 (19.1%)	
Major malformations	29 (19.1%)	19 (16.5%)	
Organ failure/organ transplant	38 (25.0%)	33 (28.7%)	
COVID-19 severity	*N* = 179		
Mild	101 (56.4%)	NA	NA
Moderate	20 (11.2%)	NA	
Severe	36 (20.1%)	NA	
Critical	22 (12.3%)	NA	

The chi-square (Χ²) test compared proportions between groups. COVID-19 severity was classified into four groups (mild, moderate, severe and critical) according to Da Costa et al. (2024) [55]. NA- not applicable

**Table 2:** MCL-1 Isoform Expression and Serum Levels in COVID-19-Positive, COVID-19-Negative, and in the Negative Control Group

Expression	COVID-19 positive	COVID-19 negative	Negative control	*p*-value
	*N* = 175	*N* = 138	*N* = 77	
MCL-1 L	2.21 ± 3.28	2.03 ± 3.85	0.63 ± 0.64	< 0.0001
MCL-1 S	9.29 ± 30.03	8.81 ± 29.53	0.12 ± 0.19	< 0.0001
MCL-1ES	22.04 ± 57.02	27.38 ± 70.75	0.60 ± 0.29	< 0.0001
Serum levels (ELISA)	*N* = 173	*N* = 142	*N* = 23	
Total MCL-1	14.073 ± 9.95	13.960 ± 9.21	3.432 ± 3.19	< 0.0001

**Table 3:** MCL-1 Isoform Expression in Subgroups According to Disease Severity

MCL-1 isoform expression	*N*	Mild	Moderate	Severe	Critical	*p*-value
COVID-19 (total)	175	99	19	35	22	
MCL-1 L		2.29 ± 3.20	3.12 ± 5.14	1.91 ± 2.92	1.57 ± 1.97	0.257
MCL-1 S		10.97 ± 31.27	21.02 ± 53.30	1.24 ± 2.56	4.35 ± 13.85	0.050
MCL-1ES		22.29 ± 48.27	46.37 ± 97.54	19.26 ± 65.74	4.28 ± 5.55	0.033
COVID-19 in girls	77	41	9	15	12	
MCL-1 L		1.61 ± 2.38	3.86 ± 7.17	1.70 ± 2.73	1.96 ± 2.50	0.360
MCL-1 S		14.28 ± 40.53	19.78 ± 56.67	1.06 ± 143	1.75 ± 3.22	0.292
MCL-1ES		20.83 ± 55.16	9.99 ± 10.18	13.17 ± 25.39	4.27 ± 5.92	0.286
COVID-19 in boys	98	58	10	20	10	
MCL-1 L		2.76 ± 3.61	2.44 ± 2.49	2.06 ± 3.11	1.10 ± 0.96	0.476
MCL-1 S		8.64 ± 22.70	22.14 ± 53.14	1.38 ± 3.18	7.47 ± 20.37	0.157
MCL-1ES		23.33 ± 43.22	79.11 ± 128.10	23.83 ± 84.89	4.29 ± 5.39	0.138
COVID-19 (0–2 y)	49	23	6	13	7	
MCL-1 L		1.21 ± 1.17	1.67 ± 1.11	1.30 ± 1.81	1.04 ± 1.09	0.856
MCL-1 S		5.43 ± 17.35	28.70 ± 69.63	0.88 ± 1.02	9.75 ± 24.45	0.515
MCL-1ES		7.99 ± 14.11	7.29 ± 6.96	3.71 ± 6.17	3.57 ± 5.42	0.258
COVID-19 (3–10 y)	69	47	3	12	7	
MCL-1 L		3.03 ± 4.01	7.71 ± 13.05	2.07 ± 2.92	2.94 ± 2.92	0.479
MCL-1 S		14.20 ± 38.54	1.81 ± 1.58	0.66 ± 0.73	3.28 ± 4.24	0.177
MCL-1ES		31.60 ± 60.54	11.80 ± 11.82	12.70 ± 22.19	6.28 ± 7.80	0.519
COVID-19 (11–18 y)	57	29	10	10	8	
MCL-1 L		1.94 ± 2.49	2.60 ± 2.38	2.51 ± 4.05	0.83 ± 0.73	0.419
MCL-1 S		10.10 ± 26.50	22.20 ± 53.14	2.41 ± 4.53	0.57 ± 0.87	0.095
MCL-1ES		18.60 ± 40.65	80.20 ± 127.60	47.30 ± 120.0	3.15 ± 3.03	0.243
COVID-19 with RV	65	41	7	9	8	
MCL-1 L		2.33 ± 9.96	5.46 ± 7.89	1.32 ± 2.16	3.01 ± 2.49	0.028
MCL-1 S		10.50 ± 30.64	27.70 ± 63.48	0.28 ± 0.21	9.64 ± 22.54	0.046
MCL-1ES		24.60 ± 49.98	55.10 ± 123.90	4.38 ± 5.34	7.95 ± 7.51	0.452
COVID-19 without RV	69	33	5	19	12	
MCL-1 L		2.42 ± 3.63	2.21 ± 1.90	2.37 ± 3.48	0.81 ± 1.02	0.496
MCL-1 S		11.50 ± 28.04	39.70 ± 74.30	1.06 ± 1.26	1.48 ± 3.26	0.033
MCL-1ES		20.90 ± 40.07	91.00 ± 119.20	11.30 ± 22.78	2.38 ± 2.70	0.036
COVID-19 with CD	149	82	18	32	17	
MCL-1 L		2.11 ± 2.83	3.27 ± 5.25	2.02 ± 3.03	1.83 ± 2.17	0.432
MCL-1 S		12.71 ± 34.10	22.16 ± 54.60	1.24 ± 2.65	5.50 ± 15.67	0.153
MCL-1ES		24.24 ± 51.63	48.93 ± 99.71	20.56 ± 68.66	4.97 ± 6.09	0.031
COVID-19 without CD	26	17	01	03	05	
MCL-1 L		3.12 ± 4.59	0.41 ± 0.00	0.69 ± 0.25	0.69 ± 0.46	0.090
MCL-1 S		2.59 ± 3.85	0.42 ± 0.00	1.31 ± 1.52	0.46 ± 0.28	0.469
MCL-1ES		12.90 ± 25.70	0.37 ± 0.00	5.40 ± 9.12	1.93 ± 2.11	0.522

Multiple comparisons of fold change values (MCL-1 isoform expression) were performed by the Kruskal–Wallis test. Data are presented as mean ± standard deviation (SD). Increased expressions are defined as those > 2.1-fold. CD = Chronic Disease; y = years; RV = respiratory virus. Significant *p*-values are highlighted in bold. Regarding RV, 134 patients were tested (65 positive and 69 negative), while 41 were not tested

**Table 4:** Serum MCL-1 Levels (ELISA) in Subgroups According to Disease Severity

MCL-1 ELISA	*N*	Mild	Moderate	Severe	Critical	*p*-value
COVID-19 (total)	173	98	19	35	21	
		14.411 ± 10.03	14.009 ± 10.35	12.049 ± 9.76	15.926 ± 9.73	0.459
COVID-19 in girls	80	43	9	16	12	
		14.258 ± 10.05	18.391 ± 9.99	10.185 ± 9.97	14.022 ± 10.79	0.096
COVID-19 in boys	93	55	10	19	9	
		14.531 ± 10.11	10.065 ± 9.44	13.618 ± 9.55	18.146 ± 7.99	0.334
COVID-19 (0–2 y)	50	24	6	14	6	
		14.950 ± 10.70	17.072 ± 9.16	8.879 ± 9.96	17.675 ± 9.68	0.139
COVID-19 (3–10 y)	67	45	4	11	7	
		15.213 ± 9.60	13.607 ± 8.60	13.262 ± 10.91	14.932 ± 11.64	0.859
COVID-19 (11–18 y)	56	29	9	10	8	
		12.721 ± 10.27	12.146 ± 12.24	15.152 ± 7.47	15.485 ± 9.13	0.861
COVID-19 with RV	68	42	8	10	8	
		15.967 ± 9.98	16.352 ± 9.73	12.759 ± 11.36	14.920 ± 10.00	0.723
COVID-19 without RV	64	31	4	18	11	
		15.672 ± 9.54	13.265 ± 13.58	11.512 ± 9.49	16.379 ± 10.31	0.276
COVID-19 with CD	148	82	18	31	17	
		15.018 ± 9.77	13.398 ± 10.29	12.412 ± 10.00	14.077 ± 9.90	0.490
COVID-19 without CD	25	16	1	4	4	
		11.301 ± 11.08	25.000 ± 0.00	9.236 ± 8.23	23.786 ± 2.43	0.154

Serum MCL-1 levels (ELISA) were compared by the Kruskal-Wallis test. Values are expressed in ng/mL. Data are presented as mean ± standard deviation (SD). CD = chronic diseases; y = years; RV = respiratory virus. Regarding RV, there were 68 positive participants, 64 negative and 41 not tested

**Table 5:** MCL-1 Isoform Expression and Serum Levels According to the Type of Chronic Disease

MCL-1 isoform	Genetic diseases	Cancer	Major malformations	Organ failure/ transplant	*p*-value
	*N* = 48	*N* = 34	*N* = 29	*N* = 38	
MCL-1 L	2.20 ± 2.66	3.15 ± 4.86	1.71 ± 2.06	1.72 ± 2.28	0.162
MCL-1 S	3.25 ± 10.50	26.24 ± 52.46	5.84 ± 19.55	9.39 ± 31.55	0.021
MCL-1ES	20.34 ± 56.76	53.01 ± 99.49	13.15 ± 24.57	11.86 ± 22.87	0.063
Serum MCL-1	*N* = 47	*N* = 35	*N* = 28	*N* = 38	
	12.869 ± 9.77	13.416 ± 9.41	14.667 ± 9.89	16.096 ± 10.37	0.548

Multiple comparisons of fold change values (MCL-1 isoform expression) were performed by the Kruskal–Wallis test. Data are presented as mean ± standard deviation (SD). Increased expressions are defined as those > 2.1-fold. Serum MCL-1 levels (ELISA) are given in ng/mL. Increased MCL-1 isoform expressions and significant *p*-values are highlighted in bold

**Table 6:** Correlations Between Serum Levels of MCL-1 and Between MCL-1 Isoform Expression

MCL-1 ELISA vs. MCL-1 isoform expression	Mild	Moderate	Severe	Critical
	*N* = 96	*N* = 18	*N* = 34	*N* = 21
MCL-1 ELISA vs. MCL-1ES	ρ 0.202	ρ -0.321* *p* = 0.20 (ns)	ρ -0.041	ρ -0.149
MCL-1 ELISA vs. MCL-1 S	ρ 0.060	ρ -0.077	ρ -0.471* *p* = 0.005	ρ 0.196
MCL-1 ELISA vs. MCL-1 L	ρ 0.133	ρ 0.123	ρ -0.270	ρ -0.011
MCL-1 ELISA vs. Ratio MCL-1 isoforms	ρ -0.067	ρ 0.394* *p* = 0.12 (ns)	ρ 0.002	ρ 0.034
MCL-1 expression (paired comparisons)	*N* = 99	*N* = 19	*N* = 35	*N* = 22
MCL-1ES vs. MCL-1 L	ρ 0.614** *p* = 0.001	ρ 0.565** *p* = 0.01	ρ 0.495* *p* = 0.003	ρ 0.599** *p* = 0.003
MCL-1 S vs. MCL-1 L	ρ 0.502** *p* = 0.01	ρ 0.398* *p* = 0.09 (ns)	ρ 0.735*** *p* = 0.001	ρ 0.684** *p* = 0.001
MCL-1ES vs. MCL-1 S	ρ 0.682** *p* = 0.001	ρ 0.330* *p* = 0.17 (ns)	ρ 0.465* *p* = 0.005	ρ 0.418* *p* = 0.05 (ns)
MCL-1 L vs. (MCL-1ES + MCL-1 S)	ρ 0.636** *p* = 0.001	ρ 0.661** *p* = 0.002	ρ 0.692** *p* = 0.001	ρ 0.824*** *p* = 0.001

MCL-1 ELISA results are presented in ng/mL, and MCL-1 isoform expressions are in fold changes values. The Spearman correlation coefficient (ρ) was applied to analyze correlations: < 0.3: no correlation; 0.30–0.50: weak; 0.51–0.70: moderate; 0.71–0.90: strong; > 0.9: very strong; ns- not significant. Correlations with significant *p*-values are marked as weak* (*p* < 0.05), moderate** (*p* < 0.01) or strong*** (*p* < 0.001)

**Table 7:** Multinomial Regression where the Reference Category is Critical COVID-19

COVID-19 severity		Parameter estimates						
		B	Error	Wald	Sig.	Exp (B)	95% CI	
							Exp (B)	
							Inferior limit	Superior limit
Mild	sMCL-1	0.009	0.027	0.113	0.737	1.009	0.957	1.065
	MCL-1ES	0.104	0.051	4.235	0.04	1.11	1.005	1.225
	MCL-1S	0.006	0.013	0.218	0.641	1.006	0.98	1.033
	MCL-1L	-0.184	0.121	2.343	0.126	0.832	0.657	1.053
	MCL-1 ratio	1.875	0.941	3.97	0.046	6.521	1.031	41.236
	Age group 0-2 years	-0.175	0.697	0.063	0.802	0.84	0.214	3.294
	3-10 years	0.116	0.659	0.031	0.86	1.123	0.309	4.087
	11-18 years	0^b^	.	.	.	.	.	.
	Sex - female	-0.238	0.542	0.193	0.66	0.788	0.272	2.279
	Sex - male	0^b^	.	.	.	.	.	.
	Without chronic disease	-0.796	0.852	0.873	0.35	0.451	0.085	2.395
	With chronic disease	0^b^	.	.	.	.	.	.
	Genetic disease	0.23	0.799	0.082	0.774	1.258	0.263	6.028
	Cancer	0.435	0.852	0.26	0.61	1.544	0.291	8.2
	Major Malformations	0.194	0.913	0.045	0.832	1.214	0.203	7.266
	Organ failure/transplant	0^b^	.	.	.	.	.	.
	With other respiratory virus	0.817	0.597	1.87	0.171	2.263	0.702	7.299
	Without other respiratory virus	0^b^	.	.	.	.	.	.
Moderate	sMCL-1	0.001	0.035	0.001	0.977	1.001	0.934	1.073
	MCL-1ES	0.105	0.051	4.315	0.038	1.111	1.006	1.227
	MCL-1S	0.005	0.014	0.113	0.737	1.005	0.977	1.033
	MCL-1L	-0.073	0.137	0.283	0.595	0.93	0.71	1.217
	MCL-1 ratio	0.948	1.211	0.613	0.434	2.58	0.24	27.688
	Age group 0-2 years	0.082	0.896	0.008	0.927	1.086	0.187	6.291
	3-10 years	-1.458	0.933	2.441	0.118	0.233	0.037	1.449
	11-18 years	0^b^	.	.	.	.	.	.
	Sex- female	-0.406	0.715	0.322	0.57	0.666	0.164	2.706
	Sex- male	0^b^	.	.	.	.	.	.
	Without chronic disease	-2.948	1.395	4.465	0.035	0.052	0.003	0.808
	With chronic disease	0^b^	.	.	.	.	.	.
	Genetic disease	-0.297	0.992	0.09	0.765	0.743	0.106	5.19
	Cancer	0.035	1.047	0.001	0.973	1.036	0.133	8.06
	Major Malformations	-1.042	1.244	0.702	0.402	0.353	0.031	4.038
	Organ failure/transplant	0^b^	.	.	.	.	.	.
	With other respiratory virus	1.115	0.835	1.782	0.182	3.049	0.593	15.671
	Without other respiratory virus	0^b^	.	.	.	.	.	.
Severe	sMCL-1	-0.007	0.031	0.055	0.815	0.993	0.934	1.056
	MCL-1ES	0.12	0.051	5.484	0.019	1.127	1.02	1.246
	MCL-1S	-0.178	0.12	2.189	0.139	0.837	0.662	1.059
	MCL-1L	-0.181	0.143	1.601	0.206	0.835	0.631	1.104
	MCL-1 ratio	2.022	0.954	4.499	0.034	7.556	1.166	48.972
	Age group 0-2 years	1.044	0.799	1.709	0.191	2.841	0.594	13.598
	3-10 years	0.116	0.778	0.022	0.881	1.123	0.245	5.159
	11-18 years	0^b^	.	.	.	.	.	.
	Sex- female	-0.143	0.64	0.05	0.823	0.867	0.247	3.037
	Sex- male	0^b^	.	.	.	.	.	.
	Without chronic disease	-1.259	1.059	1.412	0.235	0.284	0.036	2.264
	With chronic disease	0^b^	.	.	.	.	.	.
	Genetic disease	0.884	0.899	0.968	0.325	2.422	0.416	14.097
	Cancer	-0.601	1.115	0.29	0.59	0.548	0.062	4.88
	Major Malformations	-0.03	1.053	0.001	0.977	0.97	0.123	7.639
	Organ failure/transplant	0^b^	.	.	.	.	.	.
	With other respiratory virus	-0.391	0.703	0.31	0.578	0.676	0.17	2.682
	Without other respiratory virus	0^b^	.	.	.	.	.	.

b. This parameter is set to zero because it is redundant.

**Fig. 1:**
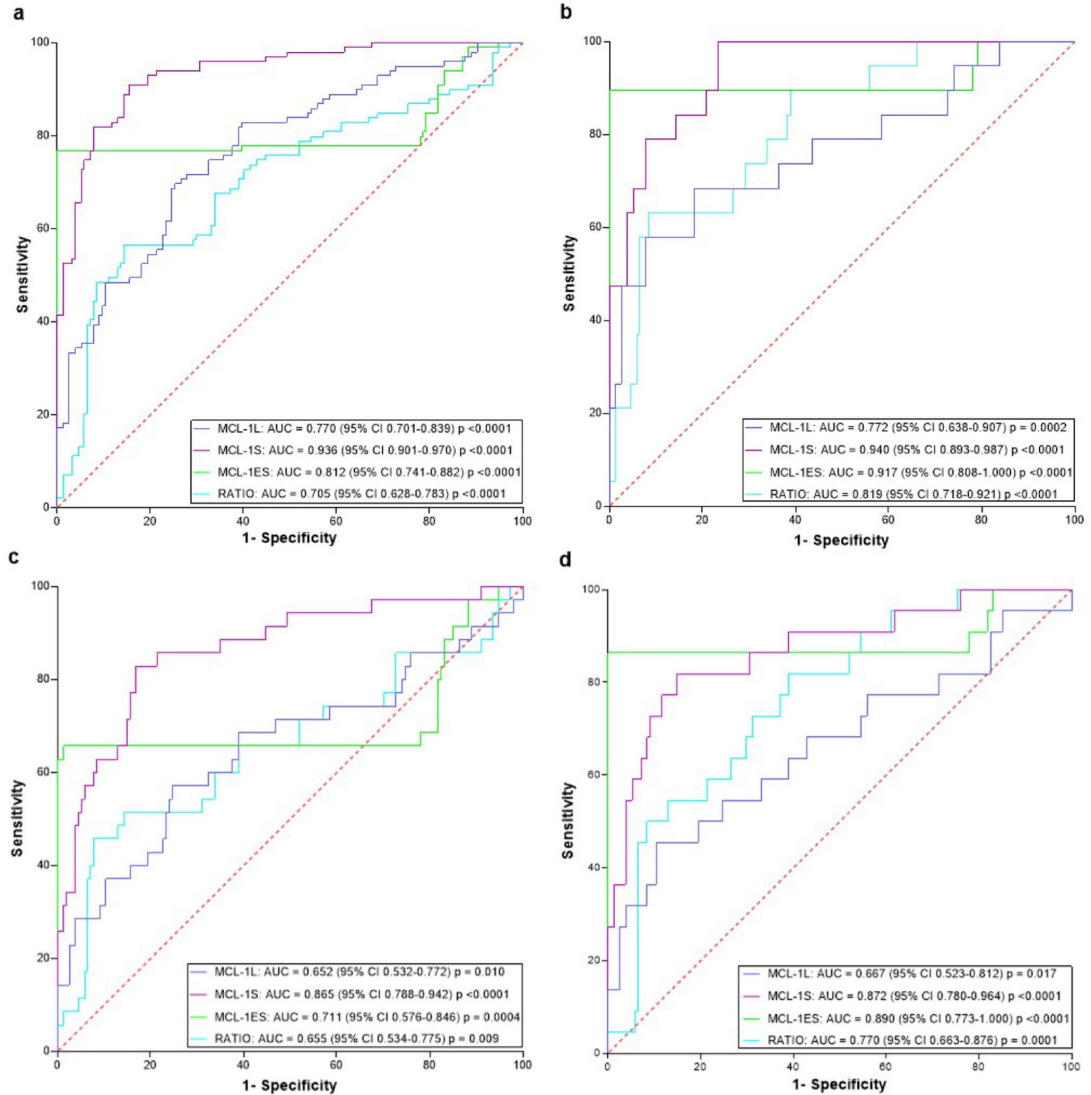
Predictive Value of MCL-1 Isoforms Using Receiver Operating Characteristic (ROC) Curves. A, B, C and D represent Mild, Moderate, Severe and Critical COVID-19

## Electronic Supplementary Material

Below is the link to the electronic supplementary material.

Supplementary Material 1

## Data Availability

All the study data (age, sex, self declared ethnicity, detection of other 19 respiratory viruses by molecular technique), as well as MCL-1 isoform expressions and MCL-1 ELISA results of participants and controls will be made available on request.

## Declarations

### Importance of the Study

Understanding why some children, particularly those with chronic medical conditions, are more likely to develop severe or critical COVID-19 remains a major gap in pediatric knowledge. This study provides the first comprehensive evaluation of MCL-1 isoform dynamics in a large cohort of unvaccinated pediatric patients with respiratory infections, revealing how SARS-CoV-2 uniquely disrupts mitochondrial apoptosis pathways. By demonstrating that specific MCL-1 isoforms and their ratio correlate strongly with disease severity and risk of clinical deterioration, this study uncovers mechanistic insights into how SARS-CoV-2 subverts mitochondrial checkpoints to evade host immunity. Importantly, these findings offer promising biomarkers that may help clinicians stratify risk early, anticipate severe progression, and tailor monitoring and treatment strategies for children, especially those with complex chronic conditions who are more vulnerable to adverse outcomes.
