# Supplementary material for: Host mitochondrial apoptotic signatures in children with chronic conditions reveal viral modulation of MCL-1 in severe SARS-CoV-2 infection
Source: Mol Biol Rep. 2026 May 9;53(1):731. doi: 10.1007/s11033-026-11885-w (PMC13157392; doi:10.1007/s11033-026-11885-w)

**Supplementary material**

**Supplementary Figure S1.** Validation of MCL-1L amplification by RT-qPCR. Amplification curves show the expected exponential increase in fluorescence across cycles for MCL-1S, indicating consistent amplification among samples. Assay performance was robust (slope = −3.449; R² = 1.00), corresponding to an amplification efficiency of 94.95%. Melting curve analysis revealed a single, well-defined peak at approximately 80.2°C, confirming amplification specificity and the absence of non-specific products or primer-dimers.


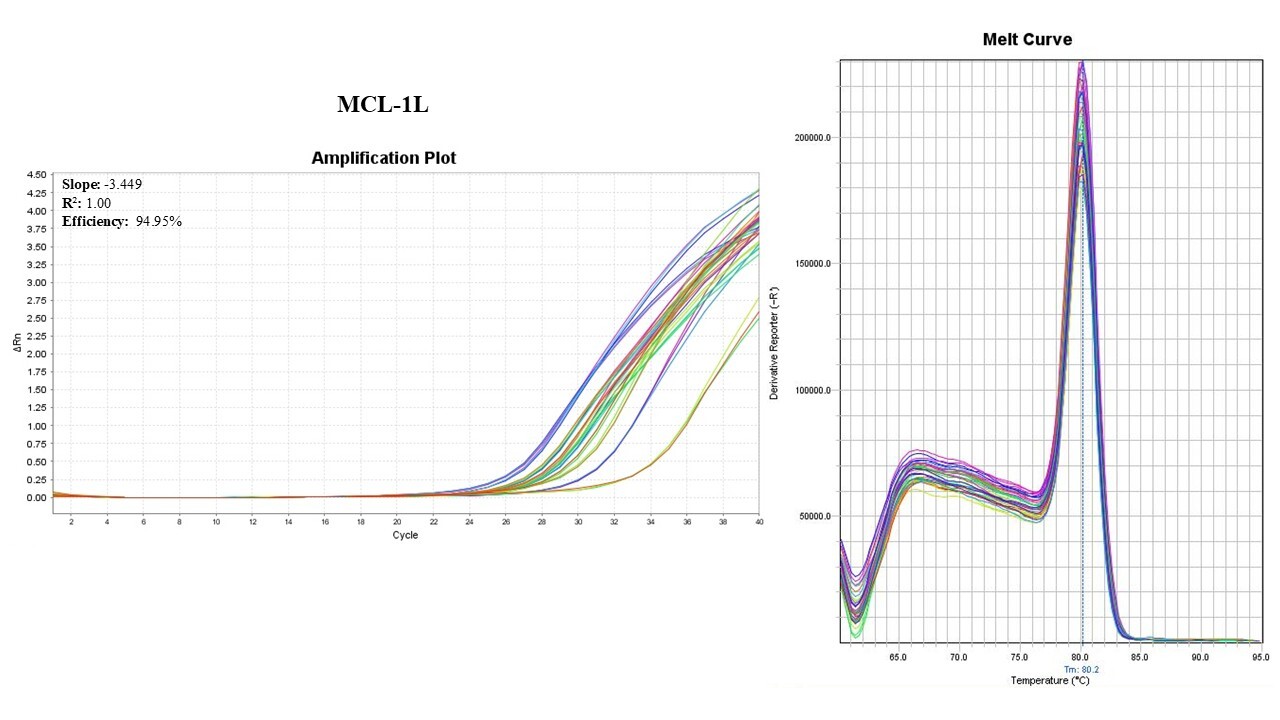


**Supplementary Figure S2.** Validation of MCL-1S amplification by RT-qPCR. Amplification curves show the expected exponential increase in fluorescence across cycles for MCL-1S, indicating consistent amplification among samples. Assay performance was robust (slope = −3.283; R² = 0.998), corresponding to an amplification efficiency of 101.64%. Melting curve analysis revealed a single, well-defined peak at approximately 75.4°C, confirming amplification specificity and the absence of non-specific products or primer-dimers.

**
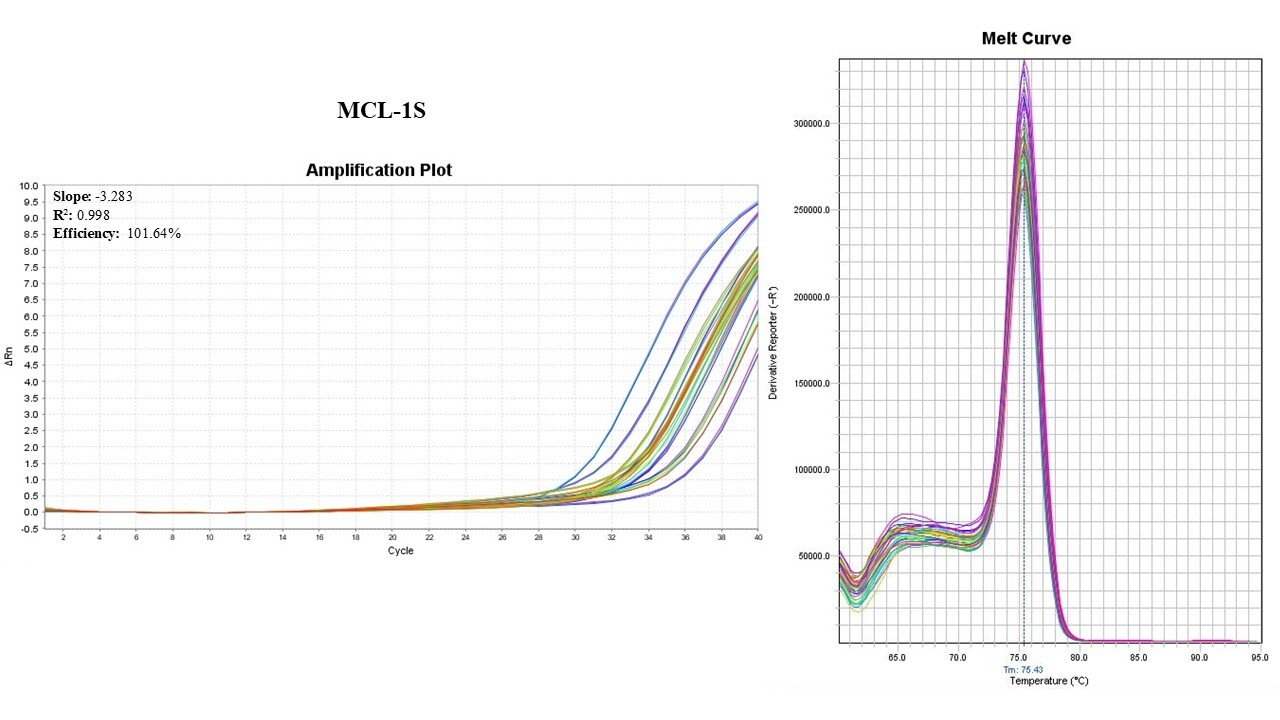
**

**Supplementary Figure S3.** Validation of MCL-1ES amplification by RT-qPCR. Amplification curves show the expected exponential increase in fluorescence across cycles for MCL-1ES, indicating consistent amplification among samples. Assay performance was robust (slope = −3.495; R² = 0.999), corresponding to an amplification efficiency of 93.25%. Melting curve analysis revealed a single, well-defined peak at approximately 75.2°C, confirming amplification specificity and the absence of non-specific products or primer-dimers.


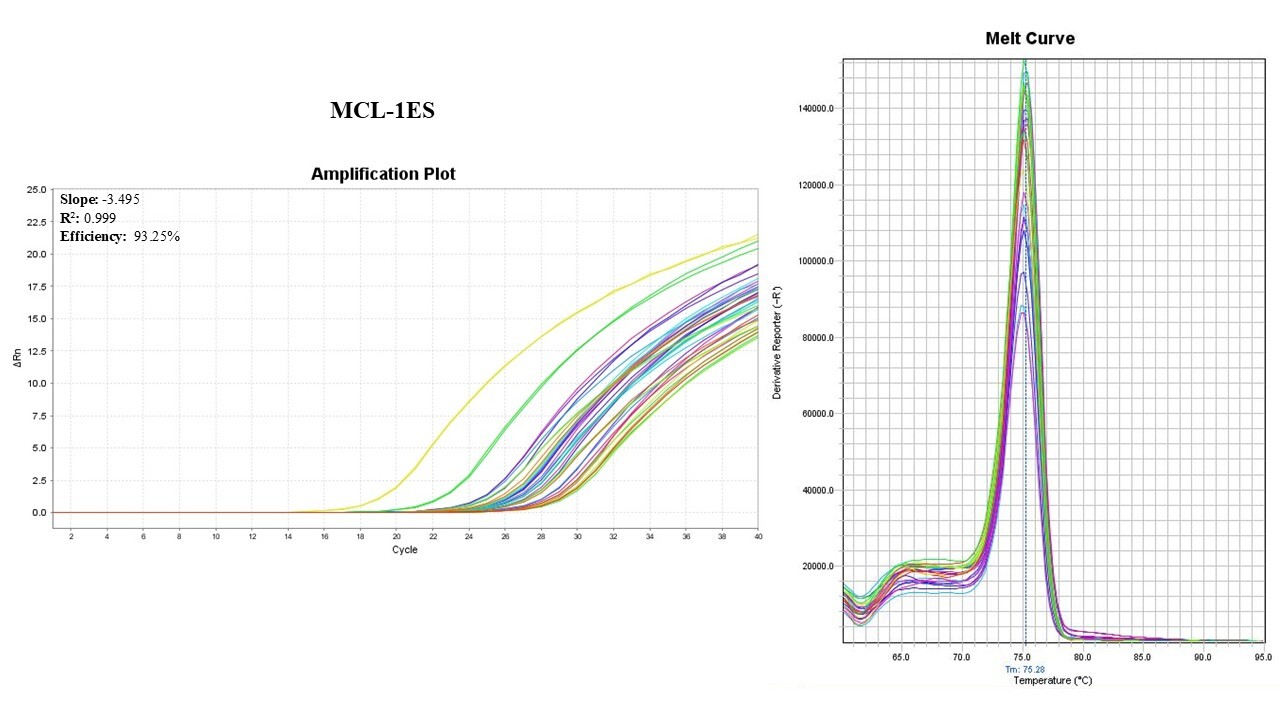

Supplement: Supplementary file 1 — Supplementary Material 1 [file 11033_2026_11885_MOESM1_ESM.docx]
